# Using Moralization as a Persuasion Strategy in Public Health Messages: A Cross-Sectional, Experimental Study on Vaping

**DOI:** 10.3390/ijerph192214859

**Published:** 2022-11-11

**Authors:** Laura Arhiri, Mihaela Alexandra Gherman, Andrei Corneliu Holman

**Affiliations:** Faculty of Psychology and Education Sciences, “Alexandru Ioan Cuza” University, Str. Toma Cozma 3, 700554 Iasi, Romania

**Keywords:** public health messages, vaping, electronic cigarettes, trust in science, attitude moralization, health behaviors, health risks, health benefits

## Abstract

Using moralization in anti-vaping public health messages as a persuasion strategy was recently recommended to address the current vaping epidemic. However, previous findings indicated this could lead to moralized attitudes in the general population, which can be very difficult to change and could severely affect social cohesion and distort risk perception. Since the safety and efficiency of using electronic cigarettes as smoking cessation devices are still being investigated, we conducted a cross-sectional, experimental study on a convenience sample of 612 Romanian never vapers, never smokers to assess how exposure to moralizing public health messages about vaping might influence their trust in future scientific results about this topic. Participants were randomized into six groups according to the type of message (“moral,” “immoral,” “neutral”) and the type of effects of vaping on smokers’ health, documented in a future fictitious study (“health benefits,” “health risks”). Results showed that the type of message moderated trust in future scientific results after controlling for participants’ general trust in science. When vaping was framed as immoral, trust in future scientific results showing health benefits was decreased, and vice versa. Implications are discussed for using moralization strategically in public health messaging to curtail or promote certain health behaviors.

## 1. Introduction

Moralization has often been employed in public health campaigns to deter people from engaging in health-risk behaviors, such as smoking [1,2,3,4,5,6,7,8,9]. The rationale behind this strategy was that shifting the focus from health implications to moral values would strengthen people’s motivation to change their behaviors in the desired direction because of the importance that human beings place on being socially perceived as moral [10]. Due to the increased prevalence of vaping and its potential negative social and health effects [11,12], it was proposed that moralization be used strategically to curtail the use of e-cigarettes in anti-vaping health campaigns [13]. However, more and more studies show that moralizing public health messages lead to attitude moralization in the general public, which can severely affect social cohesion and risk perception, suggesting that employing it in health campaigns might be more dangerous than previously thought and questioning its efficiency for the target population as well [14,15,16,17,18,19,20,21,22,23,24]. Our study aims to build upon previous results and investigate whether moralizing public health messages about vaping could threaten the general public’s trust in scientific findings.

Attitude moralization constitutes the first step in moral decision-making, one of the central fields of interest in moral psychology [25]. Several theoretical perspectives have tried to describe the temporal and spatial variations in moral values and behavior, as well as the nature of the antecedents of moral judgment. As such, intuitionists argued that people’s feelings about what may be right or wrong lead to moral judgments, with moral reasoning constituting a posthoc rationalization (e.g., [26]), while rationalists claimed that cognitive reasoning precedes moral emotions (e.g., [27,28]). Empirical data supported both claims, with moral emotions (i.e., disgust, contempt, anger) and moral cognitions contributing to assigning moral relevance to an object (e.g., [29]). Socio-cultural, intergroup, and interindividual variations in morality were usually explained with theories describing moral values or moral foundations, such as the Community-Autonomy-Divinity moral triad [7,30], subsequently reformulated as the Moral Foundations Theory [31], which argues that moralities vary on five fundamental characteristics: care, fairness, loyalty, authority, and sanctity.

The study of morality and ethics in public health has become more relevant in the wake of the COVID-19 pandemic, which shed light upon the social construction of disease, prevention, and protection efforts [32,33]. Attitude moralization is one of the most widely used persuasion strategies by public health officials for changing lifestyle health behaviors [5,6,9,21,22,23,24,34]. When employed in public health messages and campaigns, this type of strategy leads to the social and individual moralization of health behaviors in the general population, creating new social norms [5]. Despite its alleged efficiency in disease prevention [1]; but also see [19], the moralization of health behaviors in the general population can affect social cohesion, threatening societal integrity [21,22,23,24]. The health of the people who deviate from the new social norm can also be negatively affected, either through the distress associated with being stigmatized and discriminated against [2] or due to paradoxical effects associated with perceived moral reproach [33], such as maladaptive coping mechanisms (i.e., “defensive overkill” responses to perceived moral threat [23]), such as binge eating in the case of weight moralization [14]. Finally, health behavior moralization alone can increase or decrease perceived health risks, despite a lack of scientific support or even evidence to the contrary: the more (im)moral a health behavior, the more/less its associated risk [9,13].

Public trust in science refers to the extent to which people rely on scientific institutions, principles, and methods, as well as scientists when exposed to scientific knowledge [35,36,37]. Since political actors tend to rely on public opinion in their policies and decisions, public opinions should be informed by science so that scientific knowledge becomes a criterion for political decisions [38]. Moreover, a high level of public trust in science is necessary for funding certain types of research because public funding is the main source of financing in this field [39,40]. Finally, distrust in science could undermine the public’s participation in this endeavor, which is essential for its progress [41]. 

Past studies showed that trust in science could be predicted by age [42], gender [43], political affiliation [44], religiousness [45], education [46], income [42], knowledge of science [47], (social) media use [39,40,48,49]. As such, younger, liberal, non-religious people identifying as male, with a higher education, income, and previous knowledge of science, exposed to less conservative media tend to exhibit higher trust in science.

Previous research has shown that trust in science can also be associated with moralized attitudes. As such, the public’s trust in political authorities can be undermined by their science-based decisions, which contradict their moral convictions [50]. This is because moralized attitudes tend to be authority-independent, with moral duties and rights being regarded as more important than legal rules and procedures [51].

The consequences of health behavior moralization on trust in science have only recently begun to be investigated. Graso and her collaborators [52] found that the public health efforts to curtail the spreading of the new coronavirus might have moralized attitudes in the general population toward official non-pharmacological recommendations. Thus, people considered that recommendations framed with the purpose of saving lives, together with efforts in that direction, were moral. In contrast, recommendations toward revitalizing the economy and efforts toward this goal were perceived as immoral and as prioritizing profit over human lives, despite the many lives dramatically affected by shutdowns. In their second study, the authors asked participants to evaluate the quality of two research proposals using the same methodology but questioning whether efforts to eliminate COVID-19 should be continued or abandoned. Findings suggest that scientific quality was evaluated based on moral values and that attitudes toward efforts to reduce or eliminate COVID-19 have become moralized. As such, trust in science was conditioned upon attitude moralization.

Despite the above, recent studies recommend using moralization as a persuasion strategy in public health campaigns and health interventions to prevent and combat vaping [13,53]. Attitudes toward vaping have already been moralized in several socio-cultural contexts, where the general public regards it as either moral (when it is perceived as saving the lives of smokers) or immoral (when it is perceived as harmful for children and teenagers) (e.g., [4,13,53,54]). Currently, the long-term efficiency and safety of electronic cigarettes as a harm-reduction tool for quitting smoking is still debatable, with ongoing systematic reviews investigating these aspects (e.g., [55]). Thus, it would be premature to consider that vaping can save the lives of smokers. On the other side of the debate, vaping has increased among adolescents in certain contexts, but whether this increase would lead to the re-normalization of smoking is still debatable [56]. Therefore, more studies are needed for definitive answers on these topics and on the risk/benefit analysis of electronic cigarettes as efficient and safe smoking cessation tools. 

Moralized attitudes are very resistant to change, justifying almost any type of action meant to defend their underlying moral principle (for a review, see Skitka and her collaborators [20]). Given that significantly more empirical support is needed to reach a conclusion about vaping, the fact that the general population may already hold moralized attitudes toward it is concerning since these attitudes could prevent people from accepting and implementing new public health recommendations or regulations based on future scientific studies. As such, people who consider vaping to be immoral may be inclined to mistrust future empirical findings about its potential beneficial effects on smokers’ health. In contrast, people who consider vaping to be moral may be inclined to mistrust future empirical findings about its potentially harmful effects on smokers’ health and on smoking re-normalization among adolescents. The purpose of this study is to experimentally investigate these propositions because official public health messages using moralization strategically could increase the level of moralization among current moralizers [57] and diffuse it among current non-moralizers [21,22,23,24].

Based on the theoretical considerations and previous findings presented above, we formulated the following hypotheses for our study:

**Hypothesis** **1** **(H1).** *Exposure to public health messages framing vaping as moral will be associated with more trust in scientific information about the health benefits of vaping as compared to exposure to public health messages framing vaping as immoral and neutral messages.*

**Hypothesis** **2** **(H2).** *Exposure to public health messages framing vaping as immoral will be associated with less trust in scientific information about the health benefits of vaping as compared to exposure to neutral messages.*

**Hypothesis** **3** **(H3).** *Exposure to public health messages framing vaping as immoral will be associated with more trust in scientific information about the health risks of vaping as compared to exposure to public health messages framing vaping as moral and neutral messages.*

**Hypothesis** **4** **(H4).** *Exposure to public health messages framing vaping as moral will be associated with less trust in scientific information about the health risks of vaping as compared to exposure to neutral messages.*

**Hypothesis** **5** **(H5).** *Exposure to public health messages framing vaping as moral will be associated with more trust in scientific results about the health benefits of vaping than in the health risks of vaping.*

**Hypothesis** **6** **(H6).** *Exposure to public health messages framing vaping as immoral will be associated with less trust in scientific results about the health benefits of vaping than about the health risks of vaping.*

## 2. Materials and Methods

### 2.1. Participants

A convenience sample of 660 never-vapers and never-smokers was originally selected for this study through ads on social media and the personal networks of the authors, also using the snowballing sampling technique. We excluded 48 participants for the following reasons: four of them did not meet the inclusion criteria concerning time spent filling in our survey, dedicating either less than five minutes or more than 20 min to this task; 13 were excluded because they did not pass the attention checks employed. Our final sample comprised 612 people ages between 19 to 63 (*M =* 41.3, *SD =* 12.8), of whom 287 self-identified as male (46.9%) and 325—as female (53.1%). The majority were employed (76.3%), with 7.8% identifying as unemployed university students and 4.4%—as unemployed secondary school students. More than half of our participants had obtained a university degree (57.5%), having specialized in a wide array of academic disciplines (social sciences = 32.4%, humanities = 7.4%, natural sciences = 15.1%, engineering = 15.9%, life sciences = 27%). Sensitivity analyses to detect the power of the effects, which could be detected with our sample size using F-statistics and fixed effects run in G*Power, showed that, for 0.05 Alpha and 90% power, we could detect small to medium effects (f^2^ = 0.027 ≤ 0.03), following Bender and collaborators [58].

### 2.2. Study Design

We conducted a cross-sectional, experimental study with a 3 × 2 between-subjects design in which we manipulated the type of public health message presented to participants (“type of message”) and the type of scientific results about the health effects of vaping (“type of effects”). The variable “type of message” had three levels: a. “immoral” message, where we presented participants with a message according to which vaping is immoral; b. “moral” message, where we presented participants with a message according to which vaping is moral; c. neutral message, where we presented participants with a neutral text with no connection to vaping. The variable “type of effects” had two levels: a. health benefits, where participants were presented with fictitious findings of a scientific study concluding that vaping is more beneficial than harmful for smokers’ health; b. health risks, where participants were presented with fictitious findings of a scientific study concluding that vaping is more harmful than beneficial for health. The measured outcome was participants’ trust in the scientific findings with which they were presented. More details about how we operationalized our variables and for what factors we controlled are presented in Section 2.3.

### 2.3. Procedure and Instruments

First, we assessed the inclusion criteria. The never-smoker status was established if participants responded with “No” to the question “Have you ever tried cigarette smoking, even one or two puffs?” while the never-vaper status was established if participants responded with “No” to the question “Have you ever tried vaping/used an electronic cigarette, even one or two puffs?”, in line with Hammond and collaborators [59]. Participants who fulfilled these criteria were then randomly assigned to the six experimental groups, created according to the type of message to which they were exposed and to the health effects of vaping. They were told that the main purpose of our study was to explore their opinions about scientific studies on vaping [58]. A secondary purpose was enlisting their help in evaluating the visual characteristics of a public health message. To this end, they were informed that we would show them a new public health ad, which health officials planned on publicly displaying in the following months on TV and other media channels [13]. 

**Figure 1:**
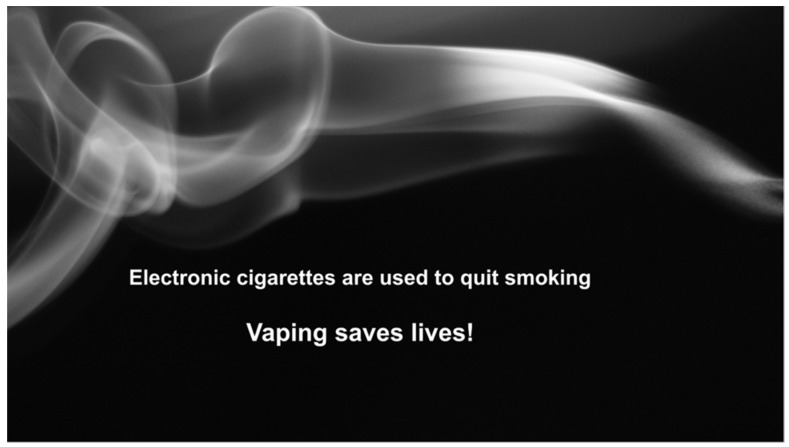
The stimulus used for the health campaign messages, which portrayed vaping as moral.

**Figure 2:**
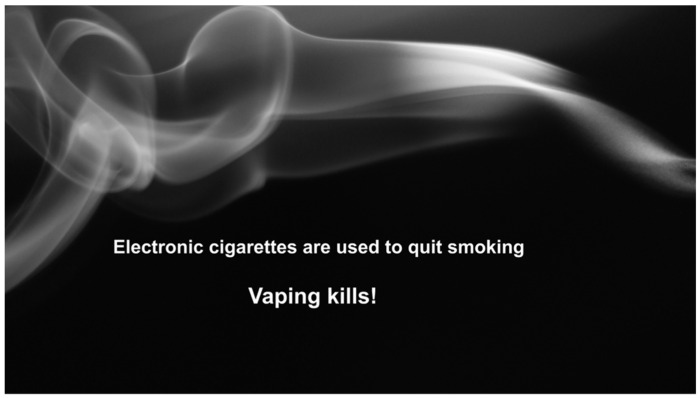
Stimulus used for the health campaign messages which portrayed vaping as immoral.

**Figure 3:**
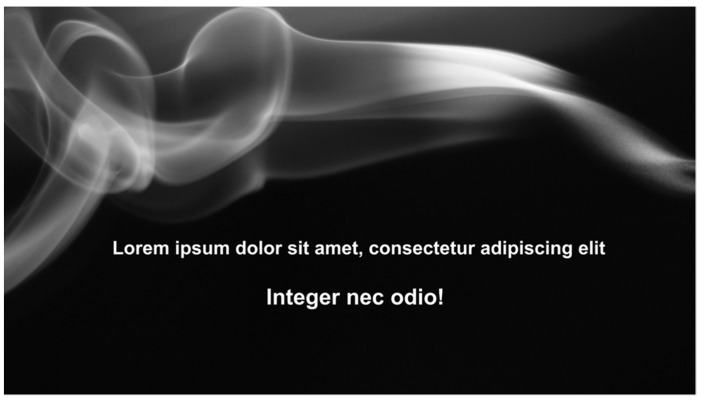
The stimulus is used for the neutral condition.

Relevant sociodemographic characteristics were assessed, in line with Altenmüller and her collaborators [60], comprising information about age, self-identified gender, education, specialization for the ones with bachelor’s and Masters’ degrees, and, respectively, for the ones with Ph.Ds. According to the group to which they had been randomly assigned, they were exposed to health campaign messages which portrayed vaping as moral (“Electronic cigarettes are used to quit smoking. Vaping saves lives!”; Figure 1), immoral (“Electronic cigarettes are used to quit smoking Vaping kills!”; Figure 2) or neutral (“Lorem ipsum dolor sit amet, con sectetur adipiscing elit Integer nec odio!”; Figure 3). 

Stimuli were created based on the images used by Minton and Gardiner [13]. The messages for the experimental groups appealed to the moral foundations of harm/care, regarded as fundamental and universal in moral psychology by most theoretical perspectives (e.g., [31,61]). The message for the control condition was a short text in Latin, a place-holder text usually employed in typesetting at least since the 1960s, without any particular meaning [62]. We chose not to use a vaping-related neutral message to the control group because we wanted to avoid any type of priming since previous research has indicated that e-cigarette use may have been moralized in the general Romanian population [54]. All participants were asked to assess, on a scale from 1 to 5, the extent to which they considered that the font type, the colors, and the background were fit for public health messages. 

As a manipulation check, we measured the extent to which their attitudes toward vaping were moralized after the experimental manipulation by adapting the instrument used by Feinberg and collaborators [29] and by us in a previous study conducted on a Romanian sample of never-vapers [54]. Since we manipulated the direction of the moralization in two of our groups (i.e., moral versus immoral) and since we wanted to avoid priming our control group with items that might have suggested moralization in either direction, we only used the first three items of the scale: “To what extent is your position on vaping a reflection of your core moral beliefs and convictions?”, “To what extent are your feelings about vaping deeply connected to your beliefs about ‘right’ and ‘wrong’?”, “To what extent do you feel the issue of vaping is a moral issue (an issue where your attitude is based on moral values)?”. Skitka and her collaborators [63] also used items similar to the first two to measure attitude moralization. From the five-item scale employed by Feinberg and collaborators [29] and by us [54], we excluded the last two items, added by Feinberg and collaborators [29] to ensure face validity, because they could only directly assess whether vaping was considered as moral or immoral, which was not applicable for our control group: “When thinking about vaping, to what extent do you ‘just know’ that it is wrong?”; “Overall, how much do you believe vaping is immoral?”. Responses ranged from 1—“Strongly disagree” to 5—“Strongly agree,” with high total scores indicating high attitude moralization (α = 0.926).

Then, participants read fictitious summaries of scientific studies concerning the health effects of vaping, adapted from Bender and collaborators [58]. The summaries kept several parameters constant to avoid confounding effects: (a). the number of words was the same in both conditions; (b). the information about the research method was the same in both conditions to ensure that the quality of the research is equivalent for all participants, regardless of the group in which they were included; (c). the outcome variable of the two studies was the same: smokers’ health; d. the results were similar, differing only in the direction of the results—beneficial or detrimental as compared to cigarette smoking. Through these texts, we manipulated the type of health effects of vaping as follows:

- Vaping is beneficial for smokers’ health

“Currently, we do not fully know the health consequences of vaping. This is why there are several research projects investigating the effects of using e-cigarettes on several health parameters. Imagine that, a year from now, a team of researchers would reach a final conclusion concerning the effects of vaping on health. Since this would be a topic of interest for many people, the summary of the study would be shared via mainstream media as well as on social media. The summary would be:

Jones and his collaborators (2023) investigated the effects of vaping on former smokers’ health. In their study, the authors compared the health status of two groups of smokers for six months. The first group, comprising 2500 smokers, had completely replaced tobacco cigarettes with electronic cigarettes. The second group continued smoking tobacco cigarettes without vaping. These two groups were the same in terms of participants’ age, initial health status, and daily nicotine consumption. The results showed stark differences in health status between the two groups. Thus, the risk of developing cancer was 95% lower in the vapers’ groups compared to the smokers’ group. Moreover, the risk of developing cardiovascular diseases also dropped by 96% in the vapers’ group compared to the smokers’ group. These results suggest that replacing tobacco cigarettes with electronic cigarettes has a positive influence on smokers’ health. In conclusion, this study brings scientific support concerning the beneficial effects on the health of vaping when e-cigarettes are used to replace tobacco cigarettes.”

b. Vaping is detrimental to smokers’ health

“Currently, we do not fully know the health consequences of vaping. This is why there are several research projects investigating the effects of using e-cigarettes on several health parameters. Imagine that, a year from now, a team of researchers would reach a final conclusion concerning the effects of vaping on health. Since this would be a topic of interest for many people, the summary of the study would be shared via mainstream media as well as on social media. The summary would be:

Jones and his collaborators (2023) investigated the effects of vaping on former smokers’ health. In their study, the authors compared the health status of two groups of smokers for six months. The first group, comprising 2500 smokers, had completely replaced tobacco cigarettes with electronic cigarettes. The second group continued smoking tobacco cigarettes without vaping. These two groups were the same in terms of participants’ age, initial health status, and daily nicotine consumption. The results showed no difference in health status between the two groups. The risk of developing cancer remained the same for vapers and for smokers. Moreover, the risk of developing cardiovascular diseases remained the same in the group of vapers as compared to the groups of smokers. These results suggest that replacing tobacco cigarettes with electronic cigarettes does not have a positive influence on smokers’ health. In conclusion, this study brings scientific support regarding the detrimental effects of vaping on smokers’ health when e-cigarettes are used to replace tobacco cigarettes”.

For this experimental manipulation, we controlled for the perceived motivation of the fictitious researchers in conducting their studies. First, we targeted potential financial conflicts of interest [64] by adding the following for both experimental conditions: “The research was conducted at several universities in the world, and none of the researchers was sponsored or financed by tobacco companies or vaping companies.”

We also controlled for the personal motivation of the researchers in choosing their topic of interest, which could affect the credibility of their results [60], by adding the following sentence for the experimental conditions: “Concerning the researchers’ motivation to conduct their studies, they declared the following: “<<We are interested in studying vaping not just for scientific reasons, but also because we, as people who don’t vape and don’t smoke, strongly believe there is a pressing social need for more scientific data concerning e-cigarette use, data which could be truly useful for everyday life>>”.

We then measured how much our participants would trust these scientific conclusions by asking them to assess the studies from the standpoint of their social value, the method employed, and, respectively, researchers’ credibility and objectivity. To this end, we used a scale adapted from Bender and collaborators [58], originally created after Nauroth and collaborators [65], comprising 13 items with answers to be provided on a Likert-type scale in 6 points (α = 0.972) (e.g., “I think that this study will generate trustworthy results.”; “I think that this study will be useful”). 

We also added a post-experimental manipulation check for the valence of the scientific results, adapted from Bender and collaborators [58]: “Please indicate the extent to which the study you just read shows that vaping has an effect on smokers’ health, from −2 Negative to +2 Positive.”

To check whether participants carefully read the text stimuli and also whether we managed to convey that the fictitious researchers did not have any conflict of interest or personal motivation to reach the respective results, we asked them to answer with “yes” or “no”/“true or false” to the following items: “The researchers declared that they smoked tobacco cigarettes”; “The researchers declared that they used electronic cigarettes,” and “The researchers did not mention anything about their statuses as smokers’ and/or vapers.”

Public engagement in science was assessed with two scales adapted after the BBVA Foundation 2011 and after Altenmüller, Lange, and Gollwitzer [60]. The first scale comprised five items assessing the frequency of their public engagement with science (α = 0.852) (e.g., “How often do you look up scientific information on the internet?”), while the second scale listed 15 potential experiences of interacting with science, which our participants might have had in the last 12 months (e.g., “I downloaded a scientific article from the internet.”; “I read a book on a scientific topic”). 

Their general trust in science was measured with the scale of science credibility [64], comprising six items with answers ranging from 1 to 6 (α = 0.912). This questionnaire measures trust in scientists, scientific results, and methods, without evaluating trust in any specific scientific field (e.g., “I am concerned by the amount of influence that scientists have in society”; “People don’t realize just how flawed a lot of scientific research really is”). Participants were rewarded with five prizes of 100 RON, offered to them based on a draw. When the experiment was finalized, participants were thanked and fully debriefed, learning about the true purpose of the study and reinforcing the fact that the scientific results presented were fictitious. 

## 3. Results

All statistical analyses were run in jamovi 2.3 [66], built upon the R infrastructure [67], with the car package [68] and GAMLj module [69].

### 3.1. Randomization Checks

Three One-Way ANOVAs with Bonferroni corrections were conducted to assess differences between our six groups of participants in their public engagement with science (frequency of their public engagement with science: F_(5, 606)_ = 0.182, *p* = 0.969; potential experiences of interacting with science: F_(5, 606)_ = 0.761, *p* = 0.578) and, respectively, in their general trust in science: F_(5, 606)_ = 1.29, *p* = 0.265.

### 3.2. Manipulation Checks

To assess the validity of the experimental manipulation of public health messages, we conducted a One-Way ANOVA with Games-Howell Post Hoc tests, with the *type of message* as the independent variable (i.e., “moral”/“immoral”/neutral) and the moralization of vaping as the outcome variable. Results supported the validity of our manipulation, with statistically significant differences between the three conditions: *Welch’s F*_(2, 400)_ = 19.8, *p* < 0.001, *est*. ω2 = 0.058. Post Hoc Tests showed that attitudes toward vaping were more moralized when participants were exposed to the “moral” message (*M =* 15.7, *SD =* 3.5) than when they were exposed to the neutral message (*M =* 13.7; *SD =* 4.66), with a statistically significant mean difference of 1.94 (*p <* 0.001). Attitudes were also more moralized when participants were exposed to the “immoral” message (*M =* 16.6, *SD =* 5.04) than to the neutral message (*M =* 13.7; *SD =* 4.66), with a statistically significant mean difference of 2.89 (*p <* 0.001). Results also showed no significant differences in attitude moralization between participants exposed to the “immoral” message (*M* = 16.6, *SD* = 5.04) and the ones exposed to the “moral” message (*M =* 15.7, *SD =* 3.5), with a non-significant mean difference of 0.96 (*p* = 0.064 > 0.05). 

To assess the validity of the experimental manipulation of the type of consequence that vaping might have on health, as described in the study to which participants were exposed, we conducted an Independent Samples *t*-Test, with “type of effects” as the independent variable (i.e., health risks vs. health benefits) and with the valence of the scientific results as the outcome variable. Participants in the health benefits condition (*M* = −0.095, *SD* = 1.03) stated that the study they had read presented findings according to which vaping was more beneficial for smokers’ health to a significantly higher degree as compared to participants in the health risks condition (*M* = 0.203, *SD* = 1.08), with a mean difference of −0.298; 95%CI = [−0.465; −0.13], which was statistically significant: t_(610)_ = −3.49, *p* < 0.001, *d* = −0.282, 95%CI = [−0.442; −0.122]. 

### 3.3. Hypotheses Testing

To test our hypotheses, we ran general linear models to test whether the association between type of effects (i.e., health risks/health benefits) and participants’ trust in science was moderated by type of message (i.e., “moral”/“immoral”/neutral). As such, the type of effects and type of message, along with their interaction, were the independent variables in our model, while trust in science was modeled as the outcome. We controlled for general trust in science, self-identified gender, and age by modeling them as independent variables as well. Education was not significantly associated with our outcome variable and was thus not included in the models. Age and general trust in science were mean-centered. Two models were tested so that all comparisons between the effects of exposure to the three types of the message could be assessed. For both models, simple contrasts were used for “type of effects,” “type of message,” and “self-identified gender,” whereby the first group, coded as 1, is compared to the subsequent groups. Health benefits were coded as 1 for the type of effects—hence, they served as the reference point for all analyses in both models. For Model 1, the neutral message was coded as 1, the “immoral” message—as 2, and the “moral” message—as 3. Thus, the reference point for our comparisons was the group exposed to the neutral message. For Model 2, “moral” messages served as reference points for group comparisons, consequently coded as 1, with “immoral” messages coded as 2 and “neutral”—as 3. Tolerance and Variance Inflation Factors suggested that collinearity was not problematic for our model (Table 1), and the distribution of the residuals did not significantly depart from normality (D = 0.05, *p =* 0.056).

Our predictors explained 42.8% of the variance for both our models (*R*^2^ = 0.428, *Adj. R*^2^ = 0.420), with interaction effects statistically significant. Control variables were not significantly associated with trust in science (self-identified gender: *F*_(1, 603)_ = 0.79, *p* = 0.374, η^2^p = 0.001; age: *F*_(1, 603)_ = 2.254, *p* = 0.134, η^2^p = 0.004; general trust in science: *F*_(1, 603)_ = 2.090, *p* = 0.149, η^2^p = 0.003).

We found statistically significant differences in trust in science according to the type of effects (*F*_(1, 603)_ = 37.118, *p* < 0.001, η^2^p = 0.058), type of message (*F*_(2, 603)_ = 4.436, *p* = 0.012, η^2^p = 0.014), and, respectively, the interaction term between them (*F*_(2, 603)_ = 194.098, *p* < 0.001, η^2^p = 0.392).

Post-hoc comparisons with Tukey corrections showed that participants exposed to “moral” messages and health benefits of vaping (*M =* 47; *SD =* 7.56) reported more trust in science than participants exposed to “immoral” messages and health benefits of vaping (*M =* 30.4; *SD =* 12.3), *t*_(603)_ = 13.13, *p_tukey_ <* 0.001, and then participants exposed to neutral messages and health benefits of vaping (*M =* 37.5; *SD =* 8.38), *t*_(603)_ = 7.33, *p_tukey_ <* 0.001, confirming H1.

Participants exposed to “immoral” messages and health benefits of vaping (*M =* 30.4; *SD =* 12.3) reported less trust in science than participants exposed to neutral messages and health benefits of vaping (*M =* 37.5; *SD =* 8.38), *t*_(603)_ = −5.76, *p_tukey_ <* 0.001, confirming H2.

Participants exposed to “immoral” messages and health risks of vaping (*M =* 53.2; *SD =* 9.91) reported more trust in science than participants exposed to “moral” messages and health risks of vaping (*M =* 34.5; *SD =* 8.15), *t*_(603)_ = 14.58, *p_tukey_ <* 0.001, and then participants exposed to neutral messages and health risks of vaping (*M =* 40.6; *SD =* 6.87), *t*_(603)_ = 9.77, *p_tukey_ <* 0.001, confirming H3.

Participants exposed to “moral” messages and health risks of vaping (*M =* 34.5; *SD =* 8.15) reported less trust in science than participants exposed to neutral messages and health risks of vaping (*M =* 40.6; *SD =* 6.87), *t*_(603)_ = −4.6, *p_tukey_ <* 0.001, confirming H4.

Participants exposed to “moral” messages and health benefits of vaping (*M =* 47; *SD =* 7.56) reported more trust in science than participants exposed to “moral” messages and health risks of vaping (*M =* 34.5; *SD =* 8.15), *t*_(603)_ = 9.53, *p_tukey_ <* 0.001, confirming H5.

Participants exposed to “immoral” messages and health benefits of vaping (*M =* 30.4; *SD =* 12.3) reported less trust in science than participants exposed to “immoral” messages and health risks of vaping (*M =* 34.5; *SD =* 8.15), *t*_(603)_ = −18.42, *p_tukey_ <* 0.001, confirming H6.

**Figure 4:**
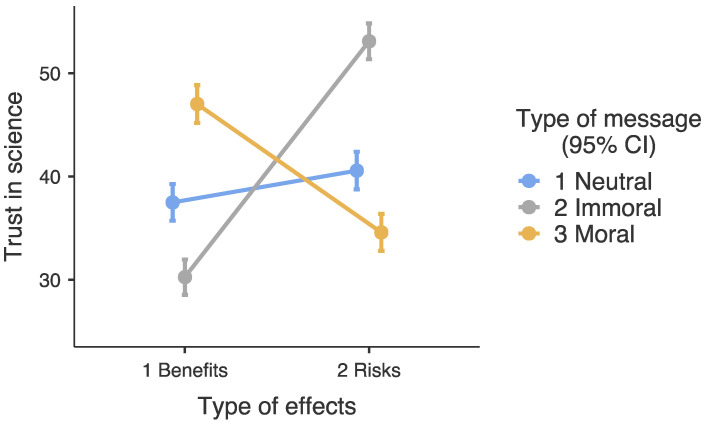
Simple slope analysis.

Simple slope analyses support the moderating role of type of message on the relationship between the type of effects and trust in science, with significant differences between participants exposed to health risks and participants exposed to health benefits according to the type of message (Table 2, Figure 4). We also found significant differences between participants exposed to health risks and participants exposed to health benefits which had been exposed to the neutral message. 

## 4. Discussion

The findings of our study suggest that exposure to moralizing public health messages in anti-vaping campaigns could undermine trust in future scientific studies, an area of investigation relevant not only to vaping, but to any health behavior or medical practice insufficiently researched so far. The public’s rejection of scientific empirical results and research methods can have deleterious effects on public health, the environment, and the economy [70,71]. Although studies report that general trust in science is still relatively high in most countries, scientific skepticism is also on the rise [72,73,74,75], with an anti-science movement gaining more and more ground [76]. This type of public discreditation of science can have severe negative outcomes on scientists, society, and daily life. The reputation of scientists can be undermined [77], and the funding allotted to certain disciplines or topics of interest could be reduced or withdrawn [78,79], negatively impacting the progress of society and jeopardizing public health [80]. 

Our results indicated that exposure to moralizing public health messages may have conditioned our participants’ trust in scientific studies. As such, after controlling for participants’ general level of trust in science, we found significant differences in their expressed trust in scientific studies, presenting future definitive conclusions about vaping according to how vaping was framed in the initial public health messages: moral or immoral. For this type of differential framing, we invoked the most agreed upon moral value among moral psychologists, harm/care, which defines immoral acts as those which cause harm and moral acts—as those which help those in need (e.g., [31,61]). Participants presented with scientific findings according to which vaping was a health risk behavior trusted those results less when they had been exposed to a public health message arguing that vaping is moral, as compared to when they had been exposed to a similar message arguing that vaping is immoral or to a neutral message. Similarly, participants presented with scientific findings according to which vaping was beneficial for smokers’ health trusted those results less when they had been exposed to a public health message arguing that vaping is immoral, as compared to when they had been exposed to a similar message arguing that vaping is moral or to a neutral message. As such, our results suggest that, in our sample, scientific findings were assessed as more or less trustworthy according to moral considerations rather than scientific merit. This is in line with previous findings about the rigidity of moralized attitudes [20] and with findings showing that trust in science may be dependent on the alignment between scientific results and people’s moral values [52]. 

We also found support for attitude moralization in the general population of Romanian never-vapers and never-smokers, in line with previous studies [13,53,54]. As such, the participants exposed to the neutral public health message and to health risks trusted our fictitious scientific results significantly more than participants exposed to the same neutral message and to health benefits. This difference cannot be attributed to moralization based solely on the results of our study. However, together with past results in the same population, we could assume that previous attitude moralization could be (also) responsible for this effect. 

Our results also support recent findings in the field of cognitive dissonance [81] about people’s preference for unfalsifiable beliefs over falsifiable ones [82]. Since moral beliefs are unfalsifiable, mental models permit adherence to them after exposure to conflicting data by offering further support for the moral mandates and disavowing any contradictory evidence (cf. [83]). The self-affirmation theory claims that cognition and behavior are guided by the motivation to preserve a general self-image of moral adequacy [84]. The self-concept may play a central role in dissonance processes, with people attempting to preserve a consistent and favorable view of themselves in terms of morality [85,86]. This could explain why our participants chose to defend their moral conviction, even when faced with scientific evidence contradicting it: feeling moral is more important to us than any other trait [10]. According to Aronson [85,87], perceiving oneself as moral constitutes one of the cognitions contributing to dissonance arousal. In other words, should our participants align with scientific evidence rather than moral mandates, their self-concept as morally good human beings would be threatened [88]. Should they have chosen to believe the scientific results, their self-esteem would have been lowered, according to the self-affirmation theory.

Our findings have theoretical and practical implications for several areas of interest. For public health, our main conclusions warn against the potential dangers of using moralization strategically in (anti-)vaping campaigns. Aside from electronic cigarettes, there are other health behaviors, medical procedures, and techniques which need further empirical testing for efficiency and safety. The most prominent examples from contemporary times are public health pharmacological and non-pharmacological recommendations about COVID-19, which were subject to change according to the newest scientific findings in the field. Trust in science was one of the most important predictors of people’s intentions to vaccinate against COVID-19 and to adhere to the non-pharmacological recommendations of public health experts, according to the findings of a recent cross-sectional study [89]. However, attitude moralization also contributed to people’s decisions about vaccination, with perceived moral reproach being significantly associated with the refusal to vaccinate [33]. For such issues, it is imperative that the people’s attitudes are sufficiently flexible to accommodate new (and sometimes contradictory) public health recommendations derived from the latest research in the field, with increased uncertainty tolerance becoming pivotal for this process [90]. 

Especially in the early days of the pandemic, public health recommendations were oftentimes contradictory, according to how the scientific results evolved, which led to mistrust in official sources of information, opening up avenues for the dissemination of overly simplistic and often conspiratorial information in the media [91]. Moralized attitudes are almost impervious to change [20], which is why using moralization strategically in public health campaigns was severely criticized in the past [21,22,23,24,34]. Moralization can lead to the replacement of the much-needed scientific arguments in the official and social discourse with publicly displayed moralistic stances and manifestations of virtue signaling [20,57,92]. Our findings add to this body of research by bringing support for the short-term effects of exposure to moralizing messages on people’s trust in consonant and, respectively, divergent subsequent scientific information. By controlling for their general trust in science, our results indicate that moralizing messages from public health officials could weigh more in discerning the credibility of the scientific information than the perceived quality of the research itself.

In relation to vaping, moralized attitudes can lead to logical fallacies, such as false dichotomies, which erroneously propose mutually exclusive courses of action—banning or commercializing electronic cigarettes to everybody [4]. The issue with these views, aside from the extreme social polarization which they may cause (e.g., [93,94]), is that they ignore the existence of in-between solutions [91]. For instance, if future studies conclude with a fair amount of certainty that the long-term consequences of vaping are less harmful than those of smoking and that vaping may help smokers quit (and not just become dual users), then they could be made available to smokers based on medical prescriptions only, and purchasable exclusively from pharmacies. If advertising them were altogether banned, the harm to adolescents could be mitigated since they would be significantly less exposed to this type of product. However, moralized attitudes would render such solutions untenable because arguments are grounded in sacred moral values rather than rational thinking: if vaping were moral, then it would be immoral to restrict access to electronic cigarettes, whereas if it were immoral then it would be immoral to allow it at all, regardless of the help they could provide to smokers [4]. In conclusion, attitude moralization could constitute a significant obstacle to developing the safest and most effective interventions to help smokers quit by eroding trust in scientific results on unrelated moral grounds. 

Our study is not without limitations. To our knowledge, this is the first study to empirically investigate with an experimental design the effects of exposure to moralizing public health messages about vaping on trust in future scientific studies. However, the effects we found were short-term; future studies should conduct longitudinal research to assess whether these effects are more or less long-lasting. Nevertheless, this type of brief public health message is usually presented frequently in mainstream media, which could increase the external validity of our findings. Longitudinal studies would help us make inferences about causality, which we cannot ascertain with a high degree of confidence based on our cross-sectional study findings. Future studies should also investigate the effects of public health messages which moralize attitudes by presenting problematic health behaviors as controllable and the ones who enact them—as uniquely responsible for curtailing them since this type of messaging is used most often [21,22,23,24]. Finally, our hypotheses should be tested on representative samples of participants to further increase external validity. Although we strived to include in our study participants of different ages and socio-economic statuses, along with a fair ratio of people self-identifying as male and, respectively, female, we did not have any participants identifying as non-binary, and we did not assess income or other variables, such as close ones have suffered from a disease associated with smoking or vaping. Future studies should take these aspects into account. 

## 5. Conclusions

Our study investigated the potential moderating effects of employing a moralizing framing in public health messages about vaping on the extent to which different types of scientific results are deemed as trustworthy by never-smokers and never-vapers. Our findings suggest that using moralization as a persuasion strategy in public health messages may decrease trust in scientific results, which contradicts the direction of moralization. As such, when participants were exposed to public health messages suggesting that vaping is moral because it improves smokers’ health, they were less inclined to trust future scientific results, which concluded the opposite. In contrast, when participants were exposed to public health messages suggesting that vaping is immoral because it adversely affects smokers’ health, they were less inclined to trust future scientific results concluding that vaping might be more beneficial for smokers’ health than tobacco cigarettes. These findings are of particular importance for health behaviors and practices which are still being investigated for safety and efficiency. Using moralization strategically by public health officials could severely impact the future adherence and acceptance of evidence-based public health recommendations. 

## Figures and Tables

**Table 1:** Parameter estimates for the two moderation models.

Variables	Effects	VIF	Tolerance	*B*	*SE*	95%CI		β	*t* ^a^	
						*LL*	*UL*			
*Model 1: “Moral” and “immoral” compared to neutral message*										
Type of message	“Immoral”/neutral	2.72	0.37	2.64	0.90	0.88	4.41	0.22	2.94	*
	“Moral”/neutral	2.65	0.38	1.77	0.92	−0.04	3.58	0.15	1.92	***
Type of effects	Health risks compared to benefits	3.07	0.33	4.50	0.74	3.05	5.95	0.38	6.10	***
Type of message * Type of effects	“Moral”/neutral * Type of effects	3.42	0.29	−15.51	1.83	−19.11	−11.91	−1.3	−8.46	***
	“Immoral”/neutral * Type of effects	3.38	0.3	19.75	1.79	16.24	23.26	1.65	11.04	***
Gender	Masculine compared to feminine	1.01	0.99	−0.66	0.74	−2.12	0.8	−0.06	−0.89	
Age		1.01	0.99	0.04	0.03	−0.01	0.10	0.05	1.50	
General trust in science		1.02	0.98	0.11	0.08	−0.04	0.26	0.05	1.45	
*Model 2: “Immoral” and neutral messages compared to “moral” messages * ^b^										
Type of message	Neutral/“Moral”	2.75	0.36	−1.77	0.92	−3.58	0.04	−0.15	−1.92	
	“Immoral”/“Moral”	2.75	0.36	0.87	0.90	−0.90	2.64	0.07	0.97	
Type of message * Type of effects	Neutral/“Moral” * Type of effects	3.34	0.30	15.51	1.83	11.91	19.11	1.30	8.46	***
	“Immoral”/“Moral” * Type of effects	3.42	0.29	35.26	1.80	31.73	38.79	2.95	19.61	***
	*F*_(8, 603)_ = 56.384, *p <* 0.001, η^2^p = 0.428									

Note: VIF = Variance Inflation Factors; *B* = unstandardized coefficients; β = standardized coefficients; ^a^ df = 603; ^b^ The coefficients for Type of the message, Self-identified gender, Age, and General trust in science in Model 2 have the same values as in Model 1; *** *p* < 0.001; * *p* < 0.05.

**Table 2:** Simple slopes analysis.

Moderator Levels	Contrast	*B*	*SE*	95%CI		β	*t*_(603)_	*F*_(1, 603)_	*p*	η²p
Type of Message	Type of Effects			*LL*	*UL*					
1 Neutral	2 Risks—1 Benefits	3.08	1.29	0.55	5.62	0.26	2.39	5.72	0.017	0.009
2 Immoral	2 Risks—1 Benefits	22.83	1.24	20.4	25.26	1.91	18.42	339.23	<0.001	0.36
3 Moral	2 Risks—1 Benefits	−12.43	1.3	−14.99	−9.87	−1.04	−9.53	90.84	<0.001	0.131

Note: *B* = unstandardized coefficients; β = standardized coefficients.

## Data Availability

The data presented in this study are available upon reasonable request from the corresponding author. The data are not publicly available due to confidentiality and anonymity guarantees given to participants in the informed consent.
